# Impairment of brain endothelial glucose transporter by methamphetamine causes blood-brain barrier dysfunction

**DOI:** 10.1186/1750-1326-6-23

**Published:** 2011-03-22

**Authors:** P M Abdul Muneer, Saleena Alikunju, Adam M Szlachetka, L Charles Murrin, James Haorah

**Affiliations:** 1 Laboratory of Neurovascular Oxidative Injury, Department of Pharmacology and Experimental Neuroscience, University of Nebraska Medical Center, Omaha, NE 68198, USA

## Abstract

**Background:**

Methamphetamine (METH), an addictive psycho-stimulant drug with euphoric effect is known to cause neurotoxicity due to oxidative stress, dopamine accumulation and glial cell activation. Here we hypothesized that METH-induced interference of glucose uptake and transport at the endothelium can disrupt the energy requirement of the blood-brain barrier (BBB) function and integrity. We undertake this study because there is no report of METH effects on glucose uptake and transport across the blood-brain barrier (BBB) to date.

**Results:**

In this study, we demonstrate that METH-induced disruption of glucose uptake by endothelium lead to BBB dysfunction. Our data indicate that a low concentration of METH (20 μM) increased the expression of glucose transporter protein-1 (GLUT1) in primary human brain endothelial cell (hBEC, main component of BBB) without affecting the glucose uptake. A high concentration of 200 μM of METH decreased both the glucose uptake and GLUT1 protein levels in hBEC culture. Transcription process appeared to regulate the changes in METH-induced GLUT1 expression. METH-induced decrease in GLUT1 protein level was associated with reduction in BBB tight junction protein occludin and zonula occludens-1. Functional assessment of the trans-endothelial electrical resistance of the cell monolayers and permeability of dye tracers in animal model validated the pharmacokinetics and molecular findings that inhibition of glucose uptake by GLUT1 inhibitor cytochalasin B (CB) aggravated the METH-induced disruption of the BBB integrity. Application of acetyl-L-carnitine suppressed the effects of METH on glucose uptake and BBB function.

**Conclusion:**

Our findings suggest that impairment of GLUT1 at the brain endothelium by METH may contribute to energy-associated disruption of tight junction assembly and loss of BBB integrity.

## Background

Methamphetamine (METH), a highly addictive drug is a potent CNS stimulant that produces euphoric effects by promoting the release of dopamine, serotonin and norepinephrine [1]. METH abuse and trafficking are increasing law enforcement and social health problems in the United States, particularly in the mid-western states where the rates of METH users among teenagers (12-17 years) and young adults (18-25 years) are highest in the country [2]. The escalating problems due to METH abuse are enormous financial and health burdens to family and society. The ability of METH to stimulate the release of dopamine rapidly from dopaminergic neurons in the reward regions of the brain produces intense euphoric effects [3]. However, acute bingeing and chronic self-administration paradigms of METH abuse cause severe neurotoxicity, monoamine deficits, hyperthermia, cardiac arrhythmia, depression, addiction, and psychiatric problems due to neuronal damage [4].

Multiple mechanisms of METH-induced neurotoxicity have been reported including hyperthermia, dopamine depletion, microglial activation, free radical formation, intrinsic cell apoptosis, and cytokine production [5,6]. Acute doses of METH produce hyperthermia that significantly contributes to neurotoxicity as a result of dopamine and intracellular METH accumulation [7,8], while chronic METH abuse causes hypothermia without an associated dopamine and serotonin depletion [8]. Interestingly, accumulation of dopamine in chronic self-administration of METH triggers the activation of microglia and loss of neurons in human brain of METH abusers [9,10]. In animals, METH-induced loss of dopaminergic neurons and decreases in dopamine levels occur in specific brain regions [1,11]. Rakic et al. (1989) demonstrated the blood-brain barrier disruption after chronic amphetamine administration in guinea pig [12]. Recent review articles describe the cellular and molecular mechanisms of METH-induced neurotoxicity as a consequence of oxidative stress, blood-brain barrier breakdown, microgliosis, and activation of the apoptotic pathway [13,14]. Disruption of mitochondrial membrane potential transition and imbalanced oxidative phosphorylation appears to regulate the oxidative stress condition and the caspase-dependent apoptosis due to chronic METH abuse [13,14]. METH abuse is also shown to exert neurotoxic effects by increasing the secretion of pro-inflammatory cytokines IL-6 and TNF-alpha in the brain [15,16].

In humans it is reported that METH abusers have severe dilated cardiomyopathy [17]. In an animal model, Treweek et al. (2007) indicated glycation of endogenous proteins and production of pro-inflammatory cytokines as possible unrecognized molecular mechanisms of cardiovascular disease in chronic METH abusers [18]. In an *in vitro *study, METH accelerates the beating rate and intracellular Ca^2+ ^oscillation pattern in rat cardiomyocytes in culture [19]. However, the underlying mechanisms of METH-elicited cardiovascular dysfunction are not well understood. In conjunction with cardiovascular damage, METH abuse appears to impair blood-brain barrier (BBB) vascular function. This includes brain hyperthermia and BBB breakdown by METH treatment [20,21]. Recently, Sharma et al. (2009) and Ramirez et al. (2009) demonstrated the oxidative damage related BBB disruption and neurotoxicity by drug of abuse [22,23].

These myriad effects of METH on cardio-neurovascular function and on astrogliosis-related neurotoxicity clearly emphasize the importance of the blood and brain interface. The blood-brain barrier, principally composed of the brain endothelium tight junction proteins, is a dynamic interface. BBB function is maintained at the expense of huge bio-energy consumption. Thus, efficient uptake and metabolism of glucose by endothelial cells regulates the selective barrier and the transport system of the interface. Importantly, the transport of glucose from the BBB into the brain regulates the energy-dependent survival of glial and neuronal cells. Brain endothelial specific glucose transporter protein 1 (GLUT1) facilitates the transport of glucose from the circulation into the brain. Brain endothelial has the highly glycosylated 55 kDa GLUT1 and the less glycosylated 45 kDa GLUT1 isoforms [24,25]. GLUT1 is localized 11% in the luminal, 45% in intracellular pool, and 44% in the abluminal side of the microvessel [26,27].

Here, we hypothesize that impairment of GLUT1 by METH at the brain endothelium can deprive glucose uptake, transport and utilization as source of energy requirement for dynamic BBB function and subsequent survival of the brain endothelial cells. We also explore the protective effect of acetyl-L-carnitine (ALC, a regulator of mitochondrial function) for prevention of GLUT1 and BBB damage from METH exposure. The primary function of ALC is transporting the long-chain fatty acids into the mitochondria for oxidation to generate ATP and it also acts as a precursor for neurotransmission. Thus, ALC is neuroprotective and often refers to as an anti-oxidant because ALC maintains the functional integrity of mitochondria by enhancing β-oxidation fatty acids for energy production [28,29].

## Results

### Effects of METH on glucose uptake and GLUT1 expression

**Figure 1:**
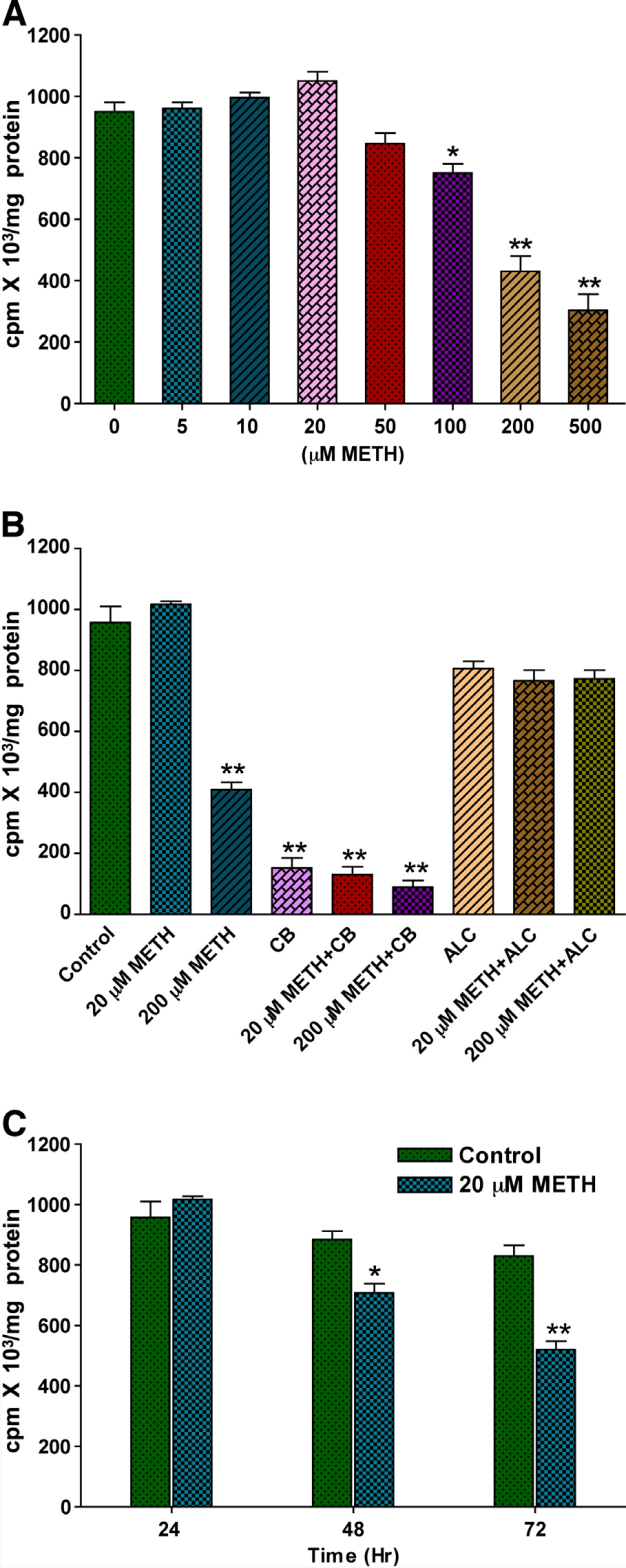
**METH inhibits glucose uptake in hBEC**. (**A**) Dose dependent Effect of METH on D-(2-^3^H)-glucose uptake in hBECs for 24 hr. (**B**) Effect of METH (20 μM and 200 μM) on D-(2-^3^H)-glucose uptake in hBECs with or without GLUT1 inhibitor cytochalasin B (CB, 10 μM) and acetyl-L-carnitine (ALC, 200 μM) for 24 hr. (**C**) Effect of METH on D-(2-^3^H)-glucose uptake in hBECs at 24, 48 and 72 hr exposure periods and compared with untreated control cells. *p < 0.05, **p < 0.01 statistically significant compared with controls.

We first examined the dose-dependent effect of METH (5 - 500 μM) exposure for 24 hr on glucose uptake by primary human brain endothelial cell (hBEC) culture. Our data indicate an insignificant increase in glucose uptake by hBECs following exposure to 5-20 μM of METH. However, the higher concentrations 50-500 μM of METH dose-dependently decreased the glucose uptake by hBECs (Figure 1A). There seemed to be a differential effect of 20 μM and 200 μM METH on glucose uptake. We examined the effects of these two METH concentrations on glucose uptake by hBECs with/without cytochalasin B (CB, GLUT inhibitor) and acetyl-L-carnitine (ALC, neuroprotective agent). As expected, CB inhibited the effect of 20 μM METH and exacerbated the decreased rate of glucose uptake by 200 μM METH, while ALC protected the effect of 200 μM METH (Figure 1B). Our time-dependent study showed that 20 μM METH gradually decreased the glucose uptake by hBECs with exposure time (Figure 1C), suggesting that low concentration of METH may activate GLUT1 in acute condition but in long-term it impairs GLUT1 function.

**Figure 2:**
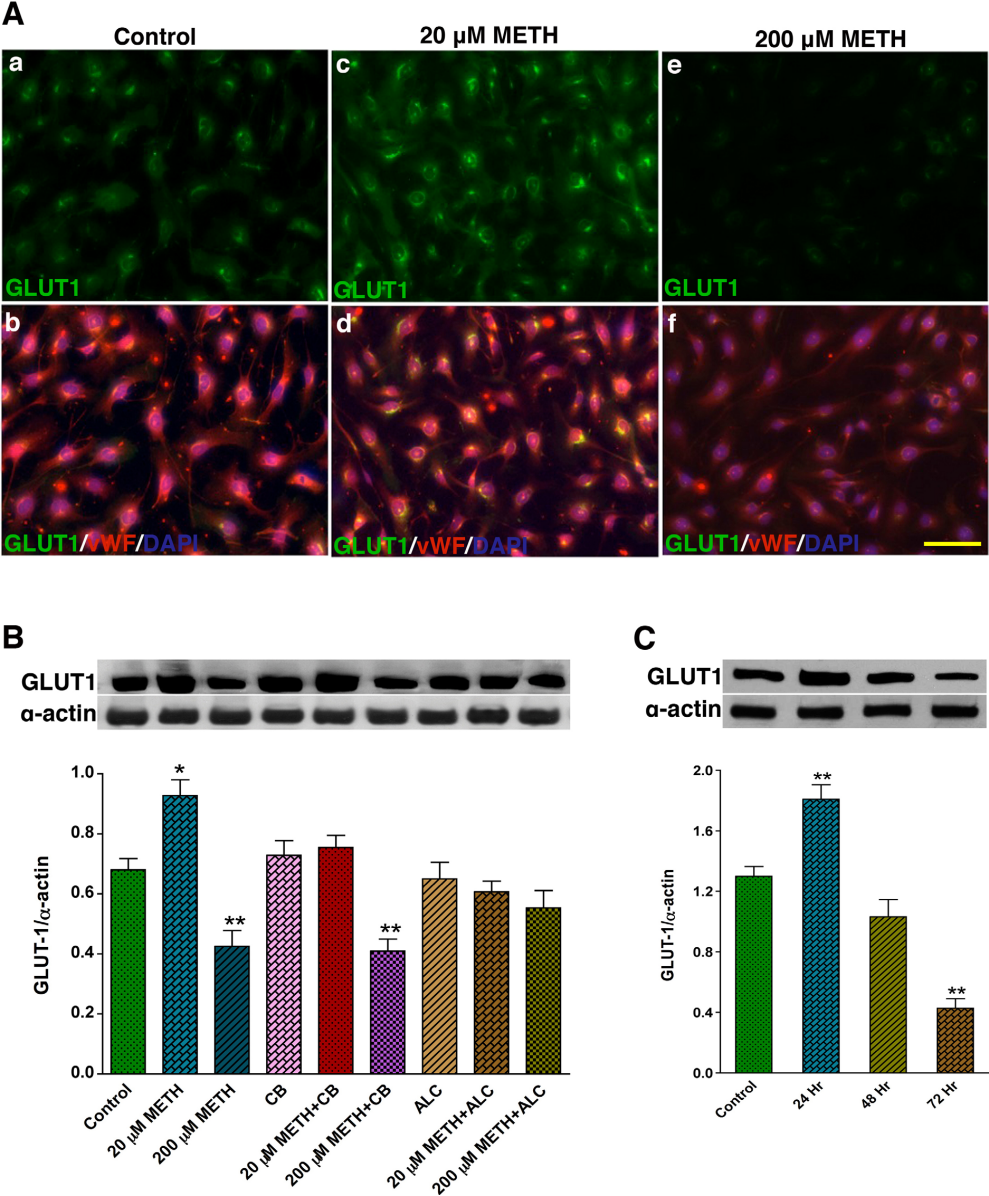
**Effect of METH on GLUT1 expression in hBEC**. (**A**) Immunocytochemical expression of GLUT1 (green) merged with von Willebrand factor (vWF, red) and DAPI (blue) in hBECs. (**B**) Effect of METH (20 μM and 200 μM) on 55 kDa isoform GLUT1 protein levels in hBEC lysate protein with different co-treatments for 24 hr. (**C**) Effect of METH (20 μM) on 55 kDa isoform GLUT1 protein levels in hBEC lysate protein with different time periods and compared with 72 hr control cells. Both bar graphs show the results, which are expressed as ratio of GLUT1 to that of α-actin bands and presented as the mean values (± SEM; n = 5). *p < 0.05, **p < 0.01 statistically significant compared with controls. Scale bar indicates 40 μm in the panels of A.

**Figure 3:**
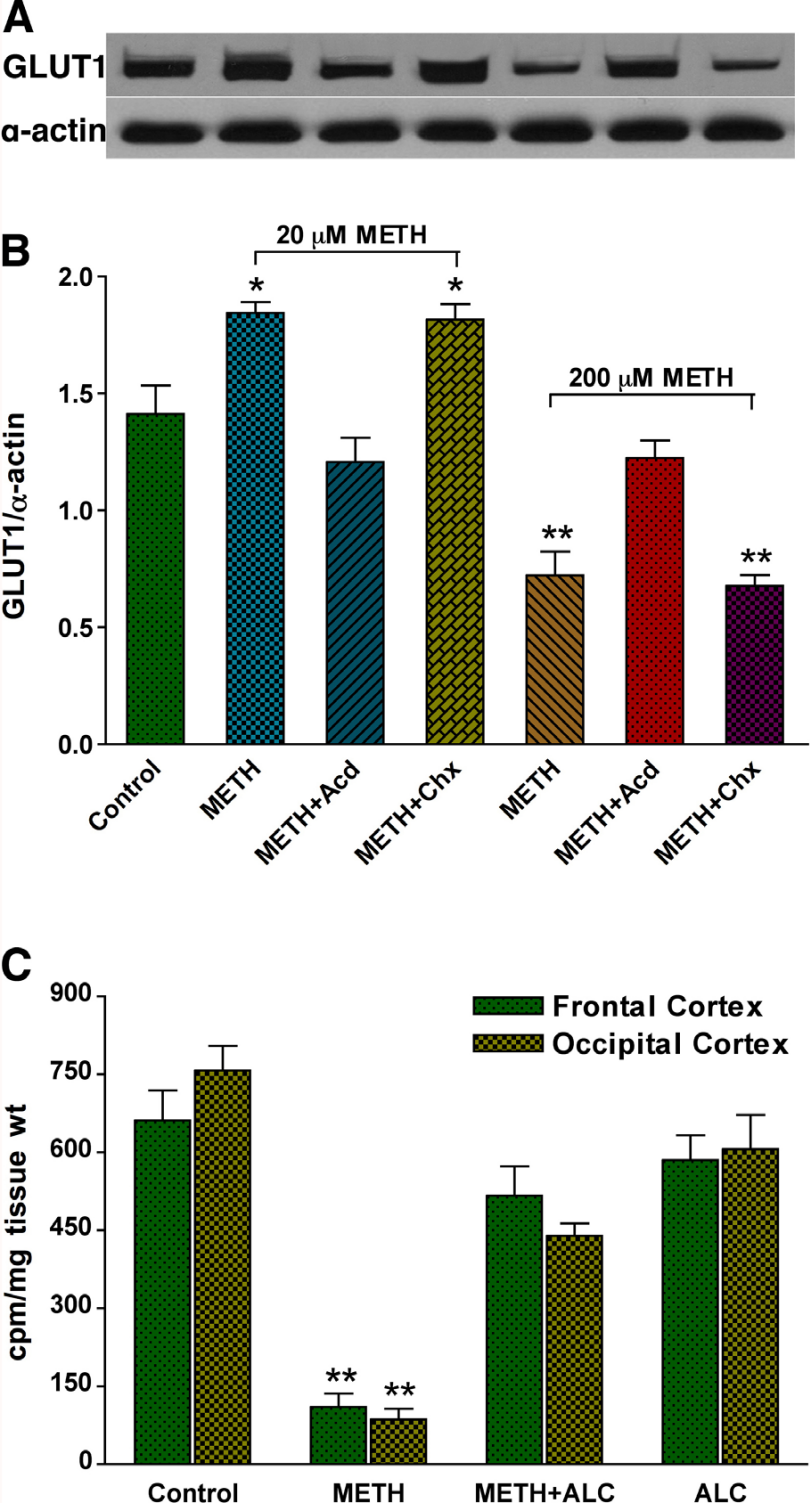
**METH inhibits glucose uptake in hBECs and animal model**. (**A-B**) Regulation of GLUT1 levels by actinomycin D (Acd, transcription inhibitor, 100 ng/mL) and cycloheximide (Chx, translation inhibitor, 10 μg/mL) in hBECs exposed to METH for 24 hr. The results show the regulation of GLUT1 was affected at transcription level. Results are expressed as ratio of GLUT1 to that of α-actin, and presented as the mean values (± SEM; n = 3). (**C**) Effect of chronic METH administration (15 mg/kg body weight) on glucose uptake *in vivo*. *Statistically significant, *p < 0.05, **p < 0.01 compared with control.

To correlate the glucose uptake results with GLUT1 protein profile, we evaluated the effect of METH on GLUT1 expression by immunocytochemistry and Western blot analyses in primary hBEC cultures. Immunocytochemistry detection indicates a substantial increase in GLUT1 expression in hBECs by 20 μM METH exposure, but a considerable decrease in GLUT1 expression by 200 μM METH exposure for 24 hr compared with untreated cells (Figure 2A a-f). Co-localization of GLUT1 protein with von Willebrand factor (vWF) indicates an intracellular localization of GLUT1 protein in brain endothelial cells. In agreement with immunocytochemical detection, Western blot analyses confirmed the increased levels of GLUT1 protein by 20 μM METH and a significant decrease in GLUT1 protein by 200 μM METH exposure compared with controls (Figure 2B). In parallel with kinetics profile of glucose uptake, exposure of 20 μM METH gradually decreased GLUT1 protein levels in hBECs for longer exposure, which validated the acute and chronic effects of low concentration of METH on GLUT1 function (Figure 2C). We noted that METH-induced alteration in GLUT1 protein levels appeared to be regulated at transcription level because actinomycin D (Acd, transcription inhibitor, 100 ng/mL) prevented the regulation of GLUT1 protein expression in 20 μM and 200 μM METH treated cells (Figure 3A-B). However, METH elicited regulation of GLUT1 expression was not affected by cycloheximide (Chx, translation inhibitor, 10 μg/mL).

### METH administration inhibits glucose uptake in animal model

To determine whether chronic administration of a moderately high dose of METH can exert a similar effect to that of high dose in cell culture, a daily dose of 15 mg/kg body weight was given to mice by i.p injection for 5-6 weeks. Then equimolar of [^3^H]-glucose (2 μCi) and unlabelled glucose was infused through the right common carotid artery as described in methods section. Our data showed that 5-6 week administration of METH almost completely inhibited glucose transport into the frontal and occipital cortex of the brain (Figure 3C). ALC protected the adverse effect of METH on glucose transport, which was also observed in the mice behavior. In that mice given the METH alone were inactive and lethargic by week 5, whereas METH+ALC administered mice were still active as the control mice.

**Figure 4:**
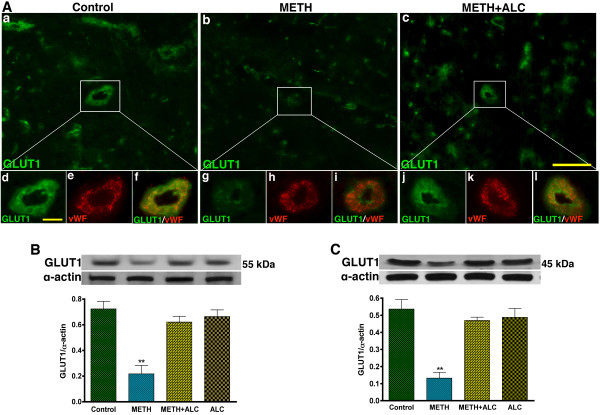
**METH exposure impairs GLUT1 expression in microvessels and brain tissues**. (**A**) Expression of GLUT1 (green) in microvessel capillaries of brain tissue section in control **(a)**, 200 μM METH **(b) **and 200 μM METH+200 μM ALC **(c)**. The expression of GLUT1 (green) merged with vWF (red) in enlarged view of a single capillary is shown in control (**d-f**), 200 μM METH **(g-i) **and 200 μM METH+200 μM ALC **(j-l)**. (**B**) Expression of 55 kDa isoform GLUT1 protein in brain microvessels and (**C**) levels of 45 kDa isoform GLUT1 protein in brain tissue protein extract. Bar graphs show the results, which are expressed as ratio of GLUT1 to that of α-actin bands and presented as the mean values (± SEM; n = 3). **Statistically significant (p < 0.01) compared with controls. Scale bar indicated 20 μm in panels of **a-c **and 5 μm (shown in **d**) in panels of **d-l**.

Staining and co-localization of GLUT1 and vWF in the brain capillaries (arterioles) sections indicated a number of positive stained for GLUT1 and vWF within the capillaries (Figure 4A a-c). Magnification of the individual capillary revealed that the diameters of these capillaries were 3-7 μm size, and also GLUT1 and vWF were localized mostly within the vascular tissue (both luminal and abluminal regions of the capillaries) with very minimal staining in the perivascular region (Figure 4A d-l). The decrease in glucose uptake correlated with the diminished level of GLUT1 expression in the microvessels of METH exposed mice. We also observed that ALC effectively protected GLUT1 expression in microvessels from chronic METH administration (Figure 4A c and 4A j-l). To confirm the compartmentalization of GLUT1 in brain microvessel (BBB) and in the brain tissues (without vessels), we extracted protein from microvessels as well as from brain tissues and subjected to Western blot analyses. METH-mediated decrease in glucose uptake and GLUT1 expression in brain microvessels correlated well with GLUT1 protein contents in protein extracts from microvessels and in brain tissue (Figure 4B-C). GLUT1 detected in protein extract microvessels was predominantly a 55 kDa isoform, whereas GLUT1 observed in protein extract from brain tissue was mostly a 45 kDa isoform with a very weak detection of 55 kDa isoform. The functional integration of this structural difference is described in the discussion section.

### METH-induced inhibition of glucose uptake impairs BBB integrity

**Figure 5:**
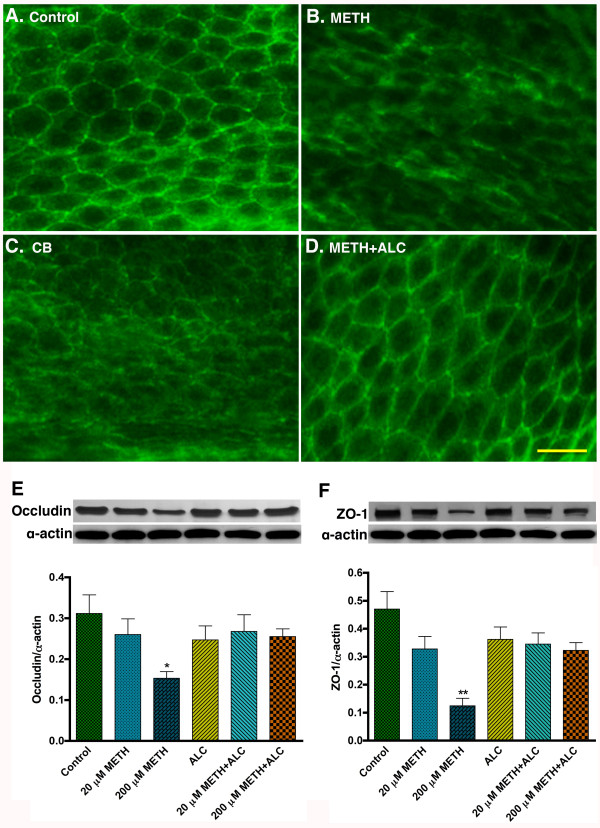
**Disruption of GLUT1 affects the integrity of BBB**. (**A-D**) Immunohistochemistry of occludin in intact brain microvessels of control (**A**), METH (15 mg/kg body weight) (**B**), CB (20 μM) (**C**) and METH+ALC (ALC = 200 μM) (**D**) treated mice. (**E**) Western blot analysis of tight junction proteins, occludin (a 65 kDa protein) and (**F**) ZO-1 (225 kDa protein) in hBECs. Bar graphs show the results that are expressed as ratio of occludin or ZO-1 to that of α-actin bands and presented as the mean values (± SEM; n = 4). Scale bar indicates 20 μm in A-D.

To evaluate the role of glucose uptake on BBB integrity, we examined the effects of METH and METH+CB on the expression of the BBB tight junction (TJ) protein occludin in intact brain microvessels. Immunohistochemistry analysis demonstrated a diminished staining of occludin with gap formation in brain microvessels from 6 weeks administration of METH compared with control (Figure 5A-B). Administration of the GLUT1 inhibitor CB alone (Figure 5C) or co-administration of with METH (data not shown) for 7 days altered the integrity of the BBB TJ protein, suggesting that efficient glucose uptake and utilization by endothelial cells play a pivotal role for proper maintenance of BBB integrity. The duration of daily CB or METH+CB administration was terminated after 7 days because the CB+METH mice were showing sign of physical weakness even if they were eating like the control mice. The staining of occludin both in control and METH+ALC brain vessels showed a sharp and continuous distribution of occludin in intact brain microvessel indicating the protective effects of ALC on BBB function (Figure 5D). Staining for ZO-1 protein demonstrated a similar pattern but not as distinct as that of occludin (data not shown). The weak staining of ZO-1 in the intact microvessels was attributed to the intracellular localization of ZO-1, which acts as the anchoring protein for occludin and claudin proteins. Alterations of occludin and ZO-1 protein levels were also evaluated by Western blot in protein extract from primary hBEC culture. We observed that both 20 μM and 200 μM of METH exposure for 24 hr reduced the levels of occludin and ZO-1 proteins in a dose-dependent manner compared with control cells (Figure 5E-F). ALC protected the effects of METH on occludin and ZO-1 protein levels. Treatment of cells with CB (inhibitor of GLUT1) completely down-regulated the levels of occludin and ZO-1 (data not shown), suggesting that glucose uptake by brain endothelial cells was essential for maintenance of BBB integrity.

### Acute and chronic METH exposure impairs BBB function

**Figure 6:**
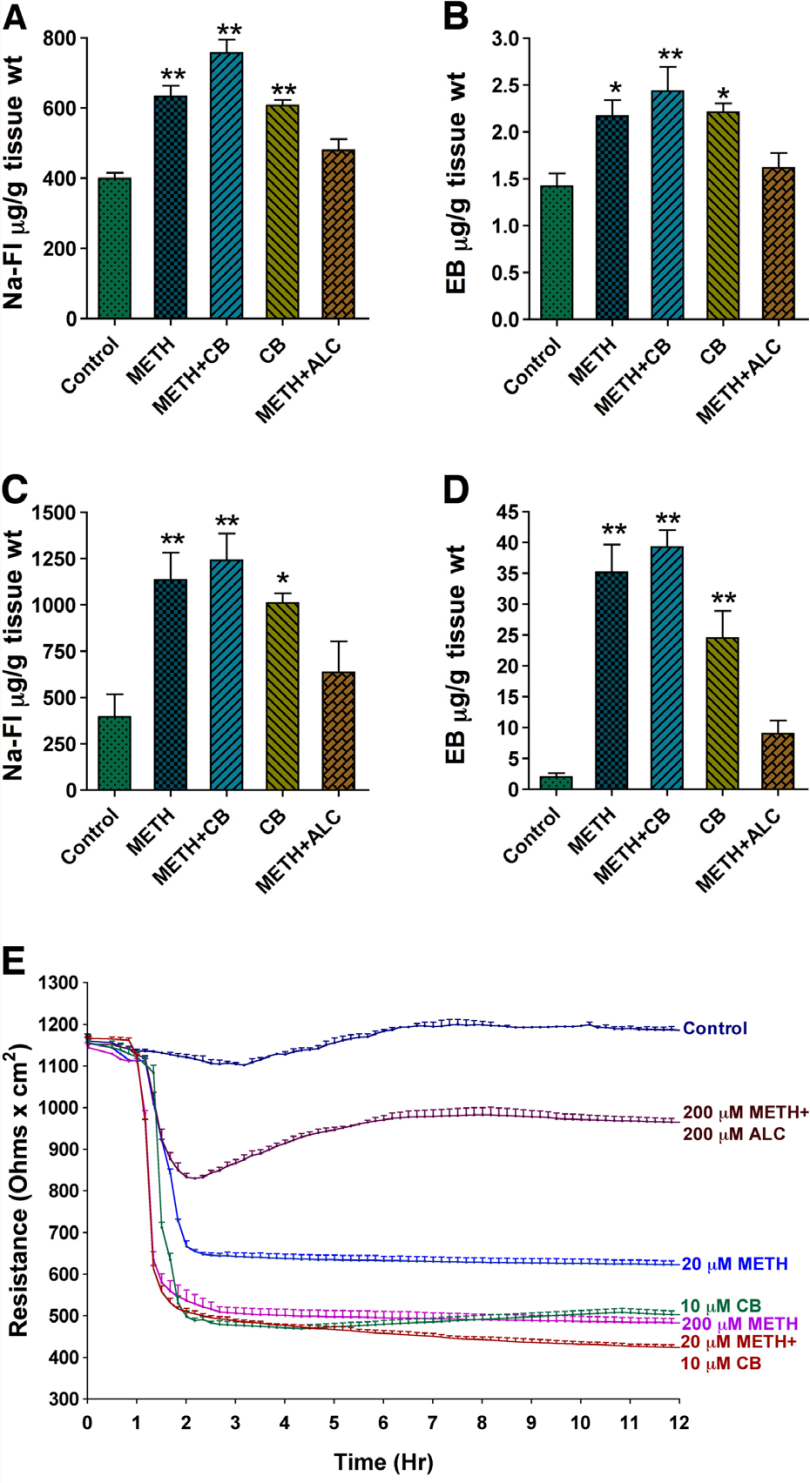
**METH and CB increase BBB permeability and decrease TEER**. **(A-D) **Permeability of sodium fluorescein (NaFl, 5 μM) or Evans Blue (EB, 5 μM) across the BBB in acute METH exposure (**A-B**) and chronic METH administration (15 mg/kg body weight) (**C-D**). (**E**) Trans-endothelial electrical resistance (TEER) of hBECs monolayers following treatment with various test compounds (n = 2). TEER was measured at 400 Hz in 10 min intervals for 12 hours. The treatments were started after stable resistance was reached for 1 hr. *Statistically significant (*p < 0.05, **p < 0.01 and ***p < 0.001) compared with controls.

To correlate the alterations of the BBB tight junction proteins, we evaluated the loss of functional integrity of the BBB by permeability and trans-endothelial electrical resistance (TEER) assays. In the *in vivo *studies, both acute (Figure 6A-B) and chronic (Figure 6C-D) exposure to METH (15 mg/kg body weight) increased the permeability of small molecular weight NaFl and large molecular weight tracer EB across the BBB compared with respective controls. Treatment of CB exacerbated the METH-elicited increase in permeability of the tracers (Figure 6A-D), suggesting that glucose uptake and metabolism play a crucial role for BBB functional integrity. To avoid contamination of tracers from microvessels, we carefully removed the microvessels from brain tissues. Thus, the dye tracers that we detected had penetrated into the brain due to BBB leakage. Our data suggest that ALC protected against METH-induced BBB permeability both in acute and in chronic conditions.

**Figure 7:**
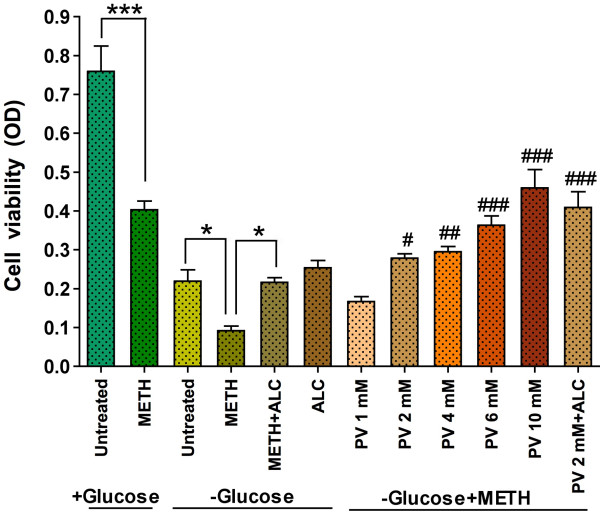
**Pyruvate protects glucose-deficient and METH induced cell dead**. Confluent hBECs cultured in 96 well plates (20000 cells/well) in normal (+ glucose) or glucose-free (-glucose) media were exposed to 100 μM METH for 48 hr, and were analyzed for cell viability assay. Untreated in (+) glucose is a positive control and in (-) glucose is a negative control. Supplementation of pyruvate (PV) in glucose-free cell culture showed dose-dependent protection from METH and glucose deficiency. *Statistically significant (*p < 0.05, and ***p < 0.001) and ^#^p < 0.05, ^##^p < 0.01 and ^###^p < 0.001 from (-) glucose+METH condition (fourth bar).

The METH-induced increase in permeability was also confirmed by a huge decrease in TEER of the BBB, which was further abrogated by addition of CB (Figure 6E). We observed that both 20 μM and 200 μM METH produced a significant reduction in electrical resistance, which was prevented by ALC. The decrease in TEER appeared gradually following METH or CB application, which culminated to a partial loss of monolayer integrity. The effect of CB and METH on TEER suggests that glucose uptake and constant energy regulation play an important role for maintaining the BBB integrity and barrier function. To support the argument, we further examined the survival of brain endothelial cells that were cultured in DMEM/F-12 glucose-free media with or without METH exposure for 48 hr. Culture of endothelial cells in normal DMEM/F-12 media with or without METH exposure was used as positive control, in which METH affected the viability of the cells. Viability of cells was significantly affected by the absence of glucose, which was further abrogated by the presence of 100 μM METH in the culture (Figure 7). Supplementation of pyruvate in glucose-free culture media does-dependently mitigated the survival of cells from the adverse effects of METH and lack of glucose. These results suggest that availability of energy substrate is very essential for survival endothelial cells and maintenance of the BBB integrity. METH appears to disrupt endothelium function by impairing GLUT1 function (i.e. decrease in glucose uptake) and perhaps glycolysis rather than affecting post-glycolysis, because the biochemical pathway for converting pyruvate to energy production seems to be unaffected. The dose-dependent protective effects of pyruvate and enhanced recuing effect of 2.0 mM pyruvate in the presence of ALC supported the notion that Krebs cycle within endothelium was still active.

## Discussion

We found that a high concentration of METH (200 μM) significantly decreased the rate of glucose uptake and glucose transporter protein-1 (GLUT1) expression. This was followed by impairment of BBB integrity as indicated by the increased permeability of the molecular tracers in an *in vivo *studies and a decrease in trans-endothelial electrical resistance in primary hBEC culture. Interestingly, a low level of METH (20 μM) insignificantly elevated both glucose uptake and GLUT1 protein expression in hBECs in acute but not in chronic condition. We demonstrated here for the first time that interference of glucose uptake and transport at the brain endothelium by chronic METH exposure lead to BBB dysfunction. METH doses of 200 μM in cell culture and 15 mg/kg body weight in animal study are considered moderately high concentrations and our low dose is close to the blood of recreational users of METH (20-40 mg/kg) [30,31]. The high dose users, who normally administer 100-1000 mg/kg may be equivalent to 100-200 μM blood levels [4]. Since 50-70% of METH is metabolized to amphetamine, 4-hydroxymeth-amphetamine and norephedrine [32,33], the levels of METH detected in the circulation represent the 30-50% un-metabolized methamphetamine.

Daily chronic intraperitoneal injection of METH (15 mg/kg body weight) in mice for 5-6 weeks almost completely inhibited the glucose transport across the BBB, which validated our *in vitro *findings. The METH-induced decrease in glucose transport across the BBB correlated with the diminished levels of GLUT1 protein expression in brain microvessels (the primary tissue components of BBB). It was interesting to find the existence of two distinct isoforms of GLUT1 at the BBB interface (microvessels) with a molecular size of 55 kDa and in the brain tissue with a molecular size of 45 kDa. Expression of these GLUT1 isoforms proteins was reduced by METH administration. Similar to these findings, it has been reported that 55 kDa GLUT1 is localized specifically in endothelial cells, whereas the 45 kDa isoform is expressed in the perivascular end-feet of astrocytes [24,25]. Functionally, the 55 kDa isoform is highly glycosylated, while the 45 kDa is the less glycosylated isoform [34]. We hypothesize that due to the dynamic nature of the BBB, the GLUT1 at this interface is mostly in the active N-glycosylated form ready to transport glucose from the peripheral circulation into the brain. The packaging of glucose into the transporter protein for delivery into the brain is possible only when GLUT1 is enzymatically glycosylated by acetylglucosamine. We suggest that once the GLUT1 (55 kDa) is translocated onto the abluminal side of the BBB, it releases the glucose at the perivascular region to the end-feet of astrocytes and neurons. Here, GLUT1 becomes mostly in the non-glycosylated 45 kDa isoform. It is possible that the weak traces of 55 kDa GLUT1 that we detected in protein extract from brain tissue may be a contamination of internal arterial vessels. Thus glycosylation of GLUT1 is crucial for functional integration and structural preservation [24].

We showed that METH-elicited inhibition of glucose uptake and suppression of GLUT1 protein at the interface were accompanied by disruption of BBB integrity. It was evident that decrease in tight junction (TJ) proteins by METH exposure led to enhanced permeability of dye/fluorescein tracers across the BBB and a huge decrease in trans-endothelial electrical resistance of the BBB. The METH-induced increase in permeability and decrease in electrical resistance across the BBB has been attributed to the toxicity of METH by some investigators [23,35-37]. Here we attributed to the impairment of glucose uptake and transport at the endothelium as one of the factors for BBB dysfunction. The rationale was that co-administration of CB exacerbated the effects of METH on TJ protein expression, BBB permeability and almost completely abrogated the BBB electrical resistance. In order to meet the constant energy demand of the dynamic BBB function, it is possible that there is synergistic interaction between GLUT1 and TJ proteins. As such, interference in endothelial glucose uptake and GLUT1 function by METH is likely to disrupt TJ protein integrity and BBB function. The question of whether such a defective BBB function will enhance passive diffusion of glucose into the brain interstitial fluid (ISF) may require further investigation. However, based on available information, BBB damage and defective in blood-brain barrier GLUT1 function seem to decrease glucose levels in the brain, similar to what we observed here. For example, in neurological diseases such as aging-related Alzheimer disease [38,39] and De Vivo disease/syndrome [40,41], where BBB disruption and GLUT1 defect were clearly observed, the levels of glucose in brain interstitial fluid were decreased from the normal values. That is because transport of glucose across the BBB into the brain interstitial fluid is exclusively an endothelium GLUT1-dependent and gradient-independent process [42,43]. Interestingly, alteration GLUT1 at the BBB has been shown to affect the facilitated entry of glucose and efflux of glucose from the brain rather than passive influx of glucose into the brain [40,43].

Glucose is the main source of energy in the brain. Therefore inhibition of glucose transport across the BBB endothelium by METH is expected to disrupt the homeostasis of glucose metabolism and energy demands of the brain cells, such as the dynamic BBB function. In part, this may explain as to why METH abusers exhibit impairment of glucose metabolism in various brain regions such as the frontal white matter [44], the striatum and thalamus [45], the limbic and paralimbic region [46], and in rat hippocampus [47]. We propose that impairment of GLUT1 function by METH exposure affects the rate of glucose uptake and metabolism (glycolysis) in brain endothelium, resulting in disruption of BBB integrity and endothelial cell death. However, METH exposure did not seem to affect the Krebs cycle because pyruvate oxidation appears to be active and normal in endothelial cells exposed to 100 μM METH. This finding suggests that the primary enzyme pyruvate dehydrogenase, which converts pyruvate to acetyl-coenzyme A was not severely damage by METH exposure. Further, stabilization of glucose uptake and GLUT1 protein levels by ALC from METH exposure may provide evidence for involvement of ALC in glucose metabolism. It is possible that ALC stabilizes the glycosylation of GLUT1 protein at the brain endothelium by donating an acetyl group to glucosamine so as to keep the acetylglucosamine functional. In this way ALC may be able to maintain the structure and functional integration of GLUT1 glycosylation at the BBB and recycle the less glycosylated GLUT1 in the neurovascular compartment. The question of whether inability of BBB to deliver normal glucose levels into the brain during METH abuse has impact on neurodegeneration is not within scope of the present study. It will require further comprehensive investigations to uncover this important issue both *in vitro *and *in vivo *studies.

## Conclusions

Our data indicate that METH mediated impairment of glucose uptake and transport at the brain endothelium disrupts the BBB function for efficient delivery of glucose into the brain. Thus, destruction of GLUT1 function at the endothelium may be a possible underlying mechanism for BBB damage.

## Methods

### Reagents

We purchased the antibodies to GLUT1 from Abcam (Cambridge, MA), antibodies to occludin and ZO-1 from Zymed (Invitrogen, Carlsbad, CA), and antibody to α-actin from Millipore (Billerica, MA). All secondary alexa fluor antibodies were purchased from Invitrogen. D-(2-^3^H)-glucose (5 mCi, 185 MBq) was purchased from PerkinElmer Life and Analytical Sciences (Waltham, MA). Cytochalasin B (CB), cycloheximide (Chx), actinomycin D (Acd) and acetyl-L-carnitine (ALC) were purchased from Sigma-Aldrich (St. Louis, MO).

### hBEC culture

Primary human brain endothelial cells (hBECs) were obtained from Dr. Persidsky's Lab, Temple University School of Medicine, and hBECs were cultured as described previously [48]. Briefly, all cell culture plates and glass cover slips were pre-coated with type 1 rat-tail collagen (0.09 mg/mL in double distilled sterile water), aspirated the excess collagen and dried the plates overnight in sterile hood. For glucose uptake and viability assays, cells were cultured in 96-well plates (20,000 cells/well), for immunohistochemistry cells were plated on 12-well glass cover slips (40,000 cells/well) and for protein extractions cells were cultured in T75 cm^2 ^flasks (1 × 10^6 ^cells/flask). DMEM/F-12 media containing 10 mM Hepes, 13 mM sodium bicarbonate (pH 7), 10% fetal bovine serum, penicillin and streptomycin (100 μg/ml each, Invitrogen) were used for cell culture. Cell culture media was changed every 3 days until tight monolayers were formed in about 6-8 days.

### *In vitro *glucose uptake

Following the modified method of Takakura [49], D-(2-^3^H)-glucose uptake was performed on hBECs cultured in 96 well plates. Cells were exposed to 20 μM and 200 μM METH for 24 hr in the presence or absence of 10 μM cytochalasin B (CB, 10 mM stock was dissolved in DMSO) or 200 μM ALC in a CO_2 _incubator. Cells were then incubated overnight in glucose-free DMEM/F-12 media containing equimolar of D-(2-^3^H)-glucose (1.0 μCi) and non-radiolabeled glucose. After washing off the excess ^3^H-glucose with phosphate saline buffer (PBS), cellular protein was precipitated with 10% TCA at 4°C for 15 min. Following the manufacturer's instruction, precipitated proteins were transferred onto a 96 well nitrocellulose filter using the Unifilter-96 well Harvester (PerkinElmer, Waltham, MA). Using the Beckman 96 well plate reader, radioactivity was measured by β-top counter. METH concentrations of 20 and 200 μM were derived from dose- and time-dependent toxicity assay (5, 10, 20, 50, 100, 200, 500 and 1000 μM of METH for 24-72 hrs), in which 20 μM had no cell toxicity effects while 200 μM of METH showed about 20% cell death after 48 hr exposure. ALC concentration of 200 μM was derived from dose-dependent study of 50-5000 μM on cell viability. Concentration of CB higher than 20 μM was toxic to hBECs (derived from 1 - 100 μM CB concentrations).

### Cell viability assay

Cell viability on hBEC was determined by 3-(4,5-dimethylthiazol-2yl)-2,5-diphenyl tetrazolium bromide (MTT) assay. The assay is based on the cleavage of yellow tetrazolium salt to purple formazan crystals by metabolically active cells. Briefly, cells were cultured in 96-well microtiter plates up to 90-100% confluent. Then the cells were treated for 48 hr in the presence or absence of METH or Pyruvate, or in combination of METH+pyruvate in glucose-free media in culture. Cells were then incubated at 37°C for 45 minutes after adding 100 μl MTT (5 mg/ml MTT in 10% FBS in 1× PBS). Then 100 μl DMSO was added just after aspirating the MTT solution and the plates were incubated at room temperature for 15 min. Absorbance of the purple formazan was detected by a microtiter plate reader at 490 nm wavelength.

### Immunfluorescent detection

For immunocytochemistry, the hBECs were cultured on glass cover slips in 12 well flasks until 80-100% confluent. Cells were then treated with 20 μM and 200 μM METH with and without CB (10 μM) or ALC (200 μM) for 24 hours. For immunohistochemistry, tissue sections (8 μm thickness) were derived from chronic METH, METH+ALC or pair-fed control mice. Cells and tissue sections were washed with PBS, fixed in acetone-methanol (1:1 v/v) fixative, blocked the cellular antigen with 3% bovine serum albumin at room temperature for 1 hr in the presence of 0.4% Triton X-100, and incubated with respective primary antibodies such as mouse anti-GLUT1 (1:250 dilution), rabbit anti-von Willebrand factor (vWF) (1:150 dilution), rabbit anti-occludin (1:250 dilution) and rabbit anti-ZO-1 (1:250 dilution) overnight at 4°C. After washing with PBS, cells/tissue sections were incubated for 1 hr with secondary antibody: anti-mouse-IgG Alexa Fluor 488 for GLUT1; anti-rabbit-IgG Alexa Fluor 594 for vWF; anti-rabbit-IgG Alexa Fluor 488 for occludin and ZO-1. Cover slips were then mounted onto glass slides with immunomount containing DAPI (Invitrogen), and fluorescence microphotographs were captured by fluorescent microscopy (Eclipse TE2000-U, Nikon microscope, Melville, NY) using NIS elements (Nikon, Melville, NY) software. GLUT1 expression was also analyzed in brain microvessels that were surgically dissected under microscope.

### Western blotting

The hBECs cultured in T-75 cm^2 ^flasks were lysed with CellLytic-M (Sigma) for 30 min at 4°C, centrifuged at 14000 g, and then total cell lysates protein concentrations were estimated by BCA (Thermo Scientific, Rockford, IL). We loaded 20 μg protein/lane and resolved the various molecular weight proteins by SDS-PAGE on gradient gels (Thermo Scientific) and then transferred the protein onto nitrocellulose membranes. After blocking, membranes were incubated for overnight with polyclonal antibody against mouse anti-GLUT1 protein (1:1000, Abcam, Cambridge, MA), rabbit anti-occludin antibody (1:250 dilution) and rabbit anti-ZO-1 antibody (1:250 dilution) at 4°C followed by 1 hr incubation with horse-radish peroxidase conjugated secondary antibodies. Immunoreactive bands were detected by West Pico chemiluminescence substrate (Thermo Scientific). Data were quantified as arbitrary densitometry intensity units using the Gelpro32 software package (Version 3.1, Media Cybernetics, Marlow, UK).

### TEER measurement

To determine the integrity of BBB function, changes in trans-endothelial electrical resistance (TEER) across the BBB were analyzed by a highly sensitive 1600R ECIS system (Applied Biophysics, Troy, NY). The ECIS system provides real-time monitoring of changes in TEER. In brief, hBECs at 20000 cells/well were plated on collagen type I coated 8W10E electrode arrays (Applied Biophysics). Once tight cell monolayers were formed, stable TEER value was monitored for 1 hr prior to treatment of cell monolayers with 20 μM and 200 μM METH in the presence or absence of CB or ALC followed by 10 hr recording of TEER at 400 hz with 10 min intervals. Confluent cell monolayers demonstrated baseline TEER readings of 1100 to 1200 Ω.cm2.

### *In vivo *glucose transport assay

Five-week old male C56/BL-6J mice purchased from Jackson Laboratory (Bar Harbor, ME) were maintained in sterile cages under pathogen-free conditions in accordance with institutional ethical guidelines for care of laboratory animals, National Institutes of Health (NIH) guidelines, and the Institutional Animal Care Use Committee. On the basis of weight matched, initially mice were grouped into control, METH and METH+ALC and they were fed the normal Lieber-DeCarli liquid-diet (Dyets, Inc. Bethlehem, PA) for 5-6 weeks. ALC was mixed in the liquid diet (1.0 mg/mL) and METH (15 mg/kg body weight) was administered daily by i.p injection for 5-6 weeks. After week 5 pair feeding of liquid-diet and monitoring the body weights, the control, METH and METH+ALC mice were anesthetized with ketamine (100 mg/kg body weight) and xylazine (10 mg/kg body weight), and equimolar of 2-^3^H-glucose (2 μCi) and unlabelled glucose in 100 μl of saline were infused through the right common carotid artery. After 1 hr, mice were euthanized and then microvessels were removed and tissues were dissected from different brain regions. Known tissue weights were homogenized with 100 μl of Krebs-Ringer phosphate-HEPES (KRPH) buffer, centrifuged at 12,000 rpm for 15 min, and 20 μl of supernatants from each condition was mixed with 4 mL of scintillation fluid. The levels of 2-^3^H-glucose in the samples were detected by liquid scintillation counter (Beckman) along with a standard curve of 2-^3^H-glucose that was run in parallel. Results were extrapolated from the standard curve and data were expressed as counts per minutes (cpm) per milligram tissue weight.

### *In vivo *BBB permeability assay

Using the sodium fluorescein (NaFl) and Evans Blue (EB) tracer dye mixtures (5 μM each), the effect of METH on BBB permeability was examined in acute and chronic animal model following an established method [50]. In acute studies, mice were anesthetized, infused with 100 μl of 200 μM of ALC or 20 μM of CB or 200 μM of METH via the common carotid artery. ALC was infused 30 min prior to METH infusion. After 1 hr of CB and METH infusion, NaFI/EB mixture was infused into the CCA and waited for another 30 min before euthanizing the animals. In chronic studies, the controls, METH (15 mg/kg body weight) and METH+ALC (ALC, 1.0 mg/mL) were given for 5-6 weeks as in glucose uptake assay. Daily i.p infusion of CB (20 μM) or CB+METH lasted for 7 days only because mice that received the CB+METH injection were getting weak by then. Mixture of NaFl/EB dyes was then infused directly into the right carotid artery as in acute studies. After decapitating, brains were removed, dissected, weighed and homogenized in 600 μl 7.5% (w/v) trichloroacetic acid (TCA). Resulting suspensions were divided into two aliquots (300 μl each). One aliquot was neutralized with 50 μl 5 N NaOH and measured by fluorimetry on a GENios microplate reader (excitation 485 nm, emission 535 nm) for NaFI determination. The other aliquot was centrifuged 10 min at 10000 rpm, 4°C, and the supernatant was measured by absorbance spectroscopy at 620 nm for EB determination. Standard curve was constructed by serial dilutions of a standard EB/NaFl solution in 7.5% TCA.

### Statistical analysis

Values are expressed as the mean ± SEM. Within an individual experiment, each data point was determined from three to five replicates. Statistical analysis of the data used GraphPad Prism V5 (Sorrento Valley, CA). Comparisons between samples were performed by one-way ANOVA with Dunnett's post-hoc test. Differences were considered significant at P values ≤ 0.05.

## Abbreviations

METH: methamphetamine; BBB: blood brain barrier; hBEC: human brain endothelial cells; GLUT: glucose transporter protein; TEER: trans endothelial electrical resistance; NaFl: sodium fluorescein; EB: Evans Blue; CB: Cytochalasin B; Chx: cycloheximide; Acd: actinomycin D; ALC: acetyl-L-carnitine; DMSO: dimethyl sulphoxide; and ZO-1: Zonula occludens-1.

## Competing interests

The authors declare that they have no competing interests.

## Authors' contributions

PMAM carried out the studies, performed the acquisition, analysis and interpretation of data and involved in manuscript preparation. SA and AMS participated in experiments. LCM contributed in proofreading the manuscript. JH designed the whole project, supervised the execution of the experiments, data interpretation and prepared the manuscript. All authors read and approved the final manuscript.

## Authors' Information

Laboratory of Neurovascular Oxidative Injury, Department of Pharmacology and Experimental Neuroscience, University of Nebraska Medical Center, Omaha, NE 68198.
